# Heat shock protein 70 (Hsp70) mediates Zika virus entry, replication, and egress from host cells

**DOI:** 10.1080/22221751.2018.1557988

**Published:** 2019-01-16

**Authors:** Sujit Pujhari, Marco Brustolin, Vanessa M. Macias, Ruth H. Nissly, Masashi Nomura, Suresh V. Kuchipudi, Jason L. Rasgon

**Affiliations:** a Department of Entomology, Center for Infectious Disease Dynamics and the Huck Institutes of the Life Sciences, The Pennsylvania State University, University Park, PA, USA; b Animal Diagnostic Laboratory, Department of Veterinary and Biomedical Sciences, The Pennsylvania State University, University Park, PA, USA; c Graduate School of Horticulture, Chiba University, Japan

**Keywords:** Arbovirus, receptor, mosquito-borne, heat shock

## Abstract

Zika virus (ZIKV) is a historically neglected mosquito-borne flavivirus that has caused recent epidemics in the western hemisphere. ZIKV has been associated with severe symptoms including infant microcephaly and Guillain-Barré syndrome, stimulating interest in understanding factors governing ZIKV infection. Heat shock protein 70 (Hsp70) has been shown to be an infection factor for multiple viruses, leading us to investigate the role of Hsp70 in the ZIKV infection process. ZIKV infection induced Hsp70 expression in host cells 48-h post-infection. Inducing Hsp70 expression in mammalian cells increased ZIKV production, whereas inhibiting Hsp70 activity reduced ZIKV viral RNA production and virion release from the cell. Hsp70 was localized both on the cell surface where it could interact with ZIKV during the initial stages of the infection process, and intracellularly where it localized with viral RNA. Blocking cell surface-localized Hsp70 using antibodies decreased ZIKV cell infection rates and production of infectious virus particles, as did competition with recombinant Hsp70 protein. Overall, Hsp70 was found to play a functional role in both the pre- and post-ZIKV infection processes affecting viral entry, replication, and egress. Understanding the interactions between Hsp70 and ZIKV may lead to novel therapeutics for ZIKV infection.

## Introduction

Zika virus (ZIKV) is a historically neglected mosquito-borne flavivirus first isolated in 1947 that, until recently, typically resulted in a handful of documented cases with mild clinical phenotypes. Beginning in 2007, larger outbreaks of the virus were first recorded, culminating in a large epidemic in the western hemisphere in 2015–2016 [1–4]. For the first time, ZIKV infection has been associated with severe symptoms including microcephaly in infants infected as fetuses, and Guillain-Barré syndrome in adults [5,6]. Considering the impacts of ZIKV on infants exposed *in utero* and its rapid spread, the World Health Organization (WHO) declared ZIKV a public health emergency of international concern [7]. The occurrence of severe clinical outcomes for fetuses and pregnant women in this outbreak has stimulated interest in determining the factors governing ZIKV infection [8,9].

The binding of a virus to specific cell surface receptor(s) is a critical step for cellular tropism and an important determinant of pathogenesis [10]. In general, flavivirus cell infection is mediated by an array of cell surface molecules and attachment cofactors [11]. Recently the role of Axl, Tyro3, and TIM1 in the pathogenesis and entry of ZIKV to the neuronal and placental cell population has been described [12–15]. However, the understanding of the ZIKV cellular infection process is still in its initial stages and needs further investigation. Heat shock protein 70 (Hsp70) has been shown to be one such factor for multiple viruses including dengue virus (DV), Japanese encephalitis virus (JEV), Hazara virus, and rotavirus, where it may act directly as a receptor or indirectly to help attach and gather viruses on the cell surface to facilitate interactions with specific high-affinity receptors [16–19]. In addition, Hsp70 plays a role in controlling viral replication in multiple virus types, including DV, influenza A virus, rabies virus and others [20–23]. Here, we demonstrate that Hsp70 is an important factor in multiple stages of the ZIKV cell infection process including viral entry, replication, and egress. Understanding the interactions between Hsp70 and ZIKV may lead to novel therapeutics for ZIKV infection.

## Results

### ZIKA virus infection induces the expression of Hsp70

**Figure 1.**
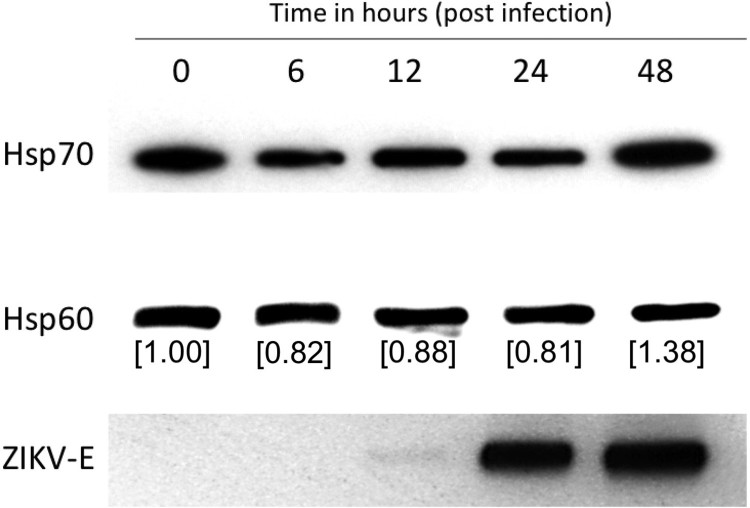
ZIKA virus induces Hsp70 protein expression. Huh7.5 cells were infected with 3 MOI ZIKV and Hsp70 assayed by western blot at 6, 12, 24 and 48 h post-infection. Hsp70 and Hsp60 bands were quantitated using ImageJ software to calculate relative Hsp70 levels. Successful virus infection in cells was determined by detection of ZIKA E protein in the cell lysate. Hsp60 was assayed as a housekeeping control.

We investigated the effect of ZIKV infection on the expression of Hsp70. Huh7.5 cells were infected with 3 MOI of the virus, and Hsp70 protein levels were measured by western blot at indicated time points. Hsp70 levels decreased in the initial timepoints following infection but increased almost 40% 48-h post-infection (Figure 1).

### Hsp70 inhibitor MKT077 reduces production of ZIKV infectious virus particles

**Figure 2.**
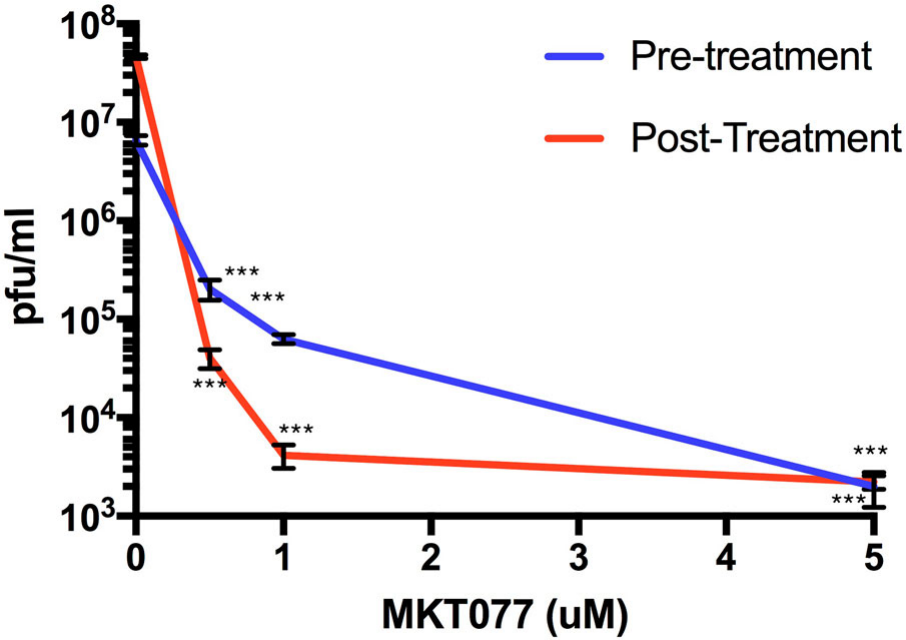
Hsp70 inhibitor MKT077 inhibits infectious ZIKV production. Huh7.5 cells were infected with 0.1 MOI of ZIKV. For the pre-treatment group, Huh7.5 cells were treated with 0.5, 1, and 5 µM MKT077 for 2 h and washed with DMEM before infection with ZIKV. For the post-treatment group, cells were infected with ZIKV, washed, and replenished with medium containing MKT077. Culture supernatants were collected 48 h post-infection. Virus titres in the culture supernatants were analysed by plaque assay. *N* = 6 per data point. Error bars denote standard error of the mean.

MKT077 is a potent allosteric inhibitor of Hsp70 that preferentially binds and inhibits the adenosine diphosphate (ADP) bound forms of Hsp70 [24]. To investigate the potential role of Hsp70 in the ZIKV infection process, we treated Huh7.5 human liver cells with MKT077. We first verified that MKT007 was not cytotoxic over the range of dosages used for our experiments (Figure S1). In the first set of experiments, we treated cells with MKT077 for 2 h before virus adsorption and then replenished the cells with maintenance medium. In the second set of experiments, cells were incubated along with MKT077 and maintenance medium after virus adsorption. After 48-h post-infection, infectious virus particles were measured in the culture supernatant. A dose-dependent reduction in the virus titre was observed for both experiments (Figure 2). The decrease in viral titre was as high as 3 logs for pre-treatment and 4 logs for post-treatment samples compared to the control, indicating that Hsp70 may have a role both at entry and post-entry levels of ZIKV infection.

### Hsp70 expression positively regulates ZIKV production

**Figure 3.**
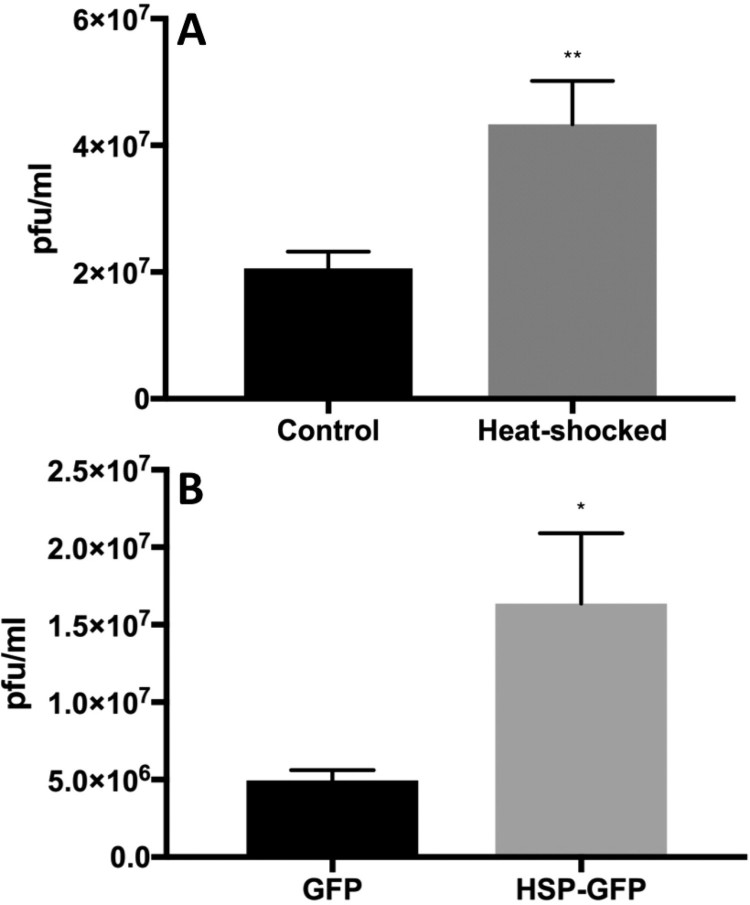
Heat shock 70 (Hsp70) protein facilitates ZIKV infection. Heat induction (A) in Huh7.5 cells and EGFP-Hsp70 overexpression (B) in 293 T cells was performed before infection of cells with ZIKV. Viral titres in harvested culture supernatants at 48-h post-infection were quantified by plaque assay. *N* = 5–6 per data point. Error bars denote standard error of the mean.

We next induced expression of Hsp70 protein in Huh7.5 cells by heat shock or in HEK 293 cells (human embryonic kidney cells) by transient overexpression using an EGFP-Hsp70 expression plasmid, infected the cells with ZIKV, and measured the production of infectious ZIKV titre in the culture supernatant 48 h after infection. Upon heat shock, Hsp70 protein levels were induced more than 3-fold at 10-h post-heat treatment compared with non-heat treated control cells (Figure S2A). For overexpressing transfected cells, we first verified that cells were expressing EGFP-Hsp70 or EGFP (control) by western blot (Figure S2). Cells were infected with ZIKV 40 h post-transfection and culture supernatants tested for virus titre at 48 h post-infection. In both heat-treated and EGFP-Hsp70 overexpressed cells, infectious ZIKV titre was significantly elevated compared to non-induced or EGFP control overexpressed cells, respectively (Figure 3).

### Role of Hsp70 in ZIKV entry process

**Figure 4.**
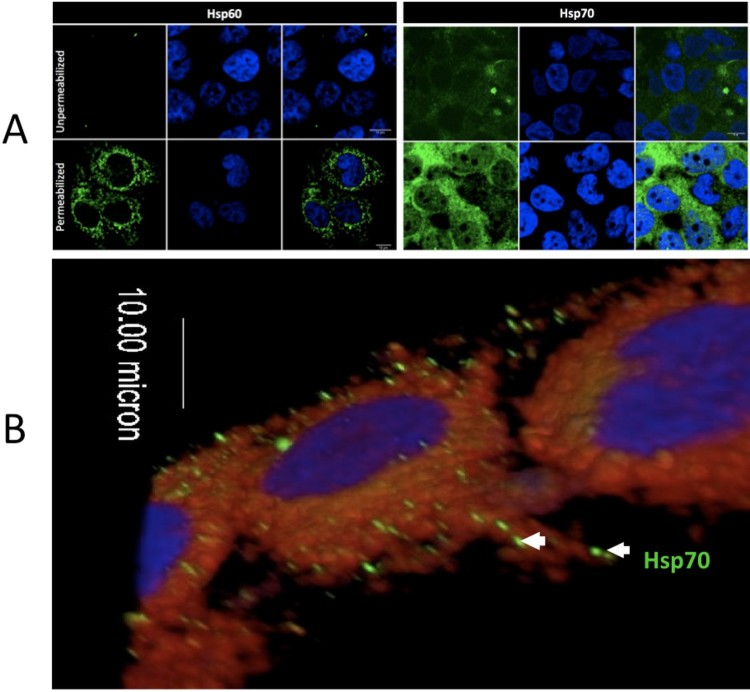
Intracellular and extracellular localization of Hsp70 in Huh7.5 cells. (A) The left panel shows staining of Hsp60 (cytoplasmic, mitochondrial protein) as a control for permeabilization and non-permeabilization staining conditions. The right panel shows the membrane and cytoplasmic staining of Hsp70. (B) 3-D confocal imaging of Hsp70 on the surface of unpermeablized Huh7.5 cells. Hsp70 = green. Cell nuclei = blue. Cell membrane = red.

Although heat shock proteins are generally thought to be cytoplasmic, Hsp70 has been localized to the surface of the cell membranes in multiple cell types [25]. We used IFA to demonstrate that Hsp70 was localized on the cell membrane surface of non-permeablized human liver cells (Huh7.5) (Figure 4).

**Figure 5.**
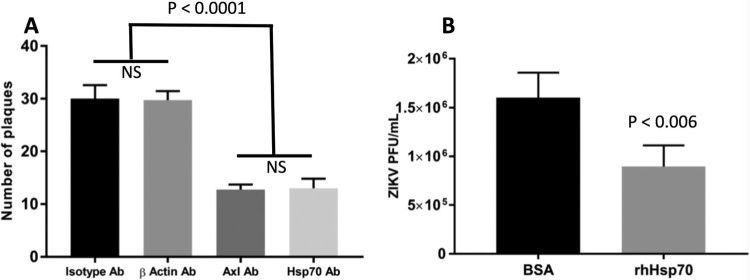
Blocking ZIKV cellular attachment to Hsp70 reduces cell infection rates and infectious particle production. (A) Antibody blocking of Hsp70 reduces Huh7.5 cellular infection with ZIKV. Axl antibody = positive control for blocking. Isotype antibody and beta-actin antibody = negative controls. After antibody treatment, cells were infected with ZIKV and plaque reduction neutralization assay performed. (B) Competition of ZIKV with recombinant human Hsp70 protein (rhHsp70) reduces Huh7.5 cellular infection with ZIKV. Control cells were treated with bovine serum albumin (BSA). Error bars denote 95% confidence intervals. NS = not significant.

If Hsp70 plays a role in ZIKV entry, using anti-Hsp70 antibody or other competitor to block the interaction between Hsp70 and ZIKV would be expected to reduce ZIKV entry. Since Vero cells are the cell line of choice for the plaque reduction neutralization assay, (gold-standard for antibody-based experiments), we focused these investigations on Vero cells [26]. Vero cells were co-incubated with antibodies to Hsp70, *β*-actin or rabbit-IgG isotype (negative control) or Axl (which has been previously demonstrated to act as a cell receptor for ZIKV [13] and which served as a positive control for the assay). Co-incubating with anti-Hsp70 antibody reduced the number of plaques by the plaque reduction neutralization assay to a similar extent as blocking Axl (Figure 5(A)).

### Competition for ZIKV attachment with recombinant Hsp70 protein reduces ZIKV infection

To further confirm the role of Hsp70 in ZIKV cellular entry, we tested whether recombinant human Hsp70 (rhHsp70) could act as a competitor for the putative cell attachment protein(s) present on the ZIKV infectious particle. ZIKV was incubated with rhHsp70 and then used to infect Huh7.5 cells, after which ZIKV production was assayed by plaque assay. Incubating ZIKV with rhHsp70 reduced the amount of infectious virus in the supernatant compared to BSA-incubated control ZIKV (Figure 5(B)).

### Role of Hsp70 in ZIKV post-entry processes

**Figure 6.**
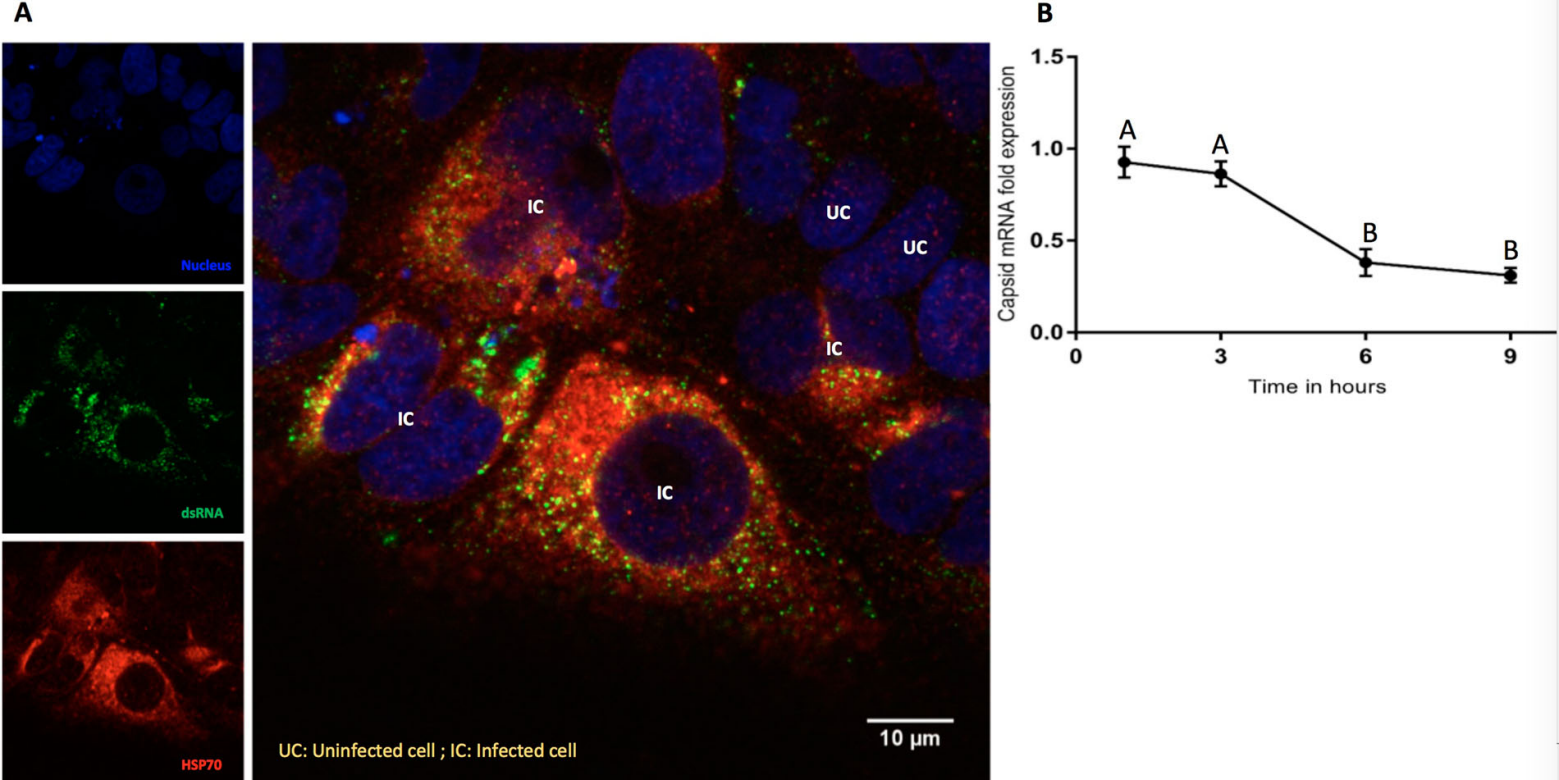
Interaction of Hsp70 and viral RNA. (A) Colocalization of intracellular Hsp70 and viral RNA. ZIKA virus infected Huh7.5 cells were fixed at 12-h post-infection. Cells were stained for dsRNA (green) and Hsp70 (red). Cell nuclei were stained with Hoechst 33342 (blue). Yellow dots indicate co-localized dsRNA and Hsp70. IC = ZIKV infected cell. UC = ZIKV uninfected cell. (B) Relative quantitation of ZIKA virus capsid mRNA by real-time RT-PCR in the presence of MKT077. Cells were infected with 6 MOI of ZIKV in the presence of MKT077. The *Y*-axis represents the expression fold-change of viral capsid mRNA normalized to vehicle (DMSO) control. Treatments with different letters are statistically different (*P* < .01).

Interaction between Hsp70 and viral RNA: We used confocal microscopy to detect the viral replication complex in infected cells using an antibody specific for dsRNA (a viral replication intermediate). Confocal image analysis demonstrated elevated Hsp70 expression in infected relative to uninfected cells and co-localization of dsRNA and Hsp70, suggesting that Hsp70 is a part of the virus replication complex (Figure 6(A)).

Effect of Hsp70 on viral RNA synthesis: We next tested whether the reduction of virus titre in the presence of Hsp70 inhibitor (Figure 2) could be explained by inhibition of viral genome transcription. We analysed the effect of MKT077 on viral mRNA synthesis. Total RNA was extracted from the untreated infected Huh7.5 cells and MKT077-treated infected cells harvested at different time points (1, 3, 6, and 9 h) after infection with ZIKV. In the presence of MKT077, a time-dependent reduction in the viral capsid mRNA was observed (Figure 6(B)), indicating that MKT077 impaired viral transcription.

**Figure 7.**
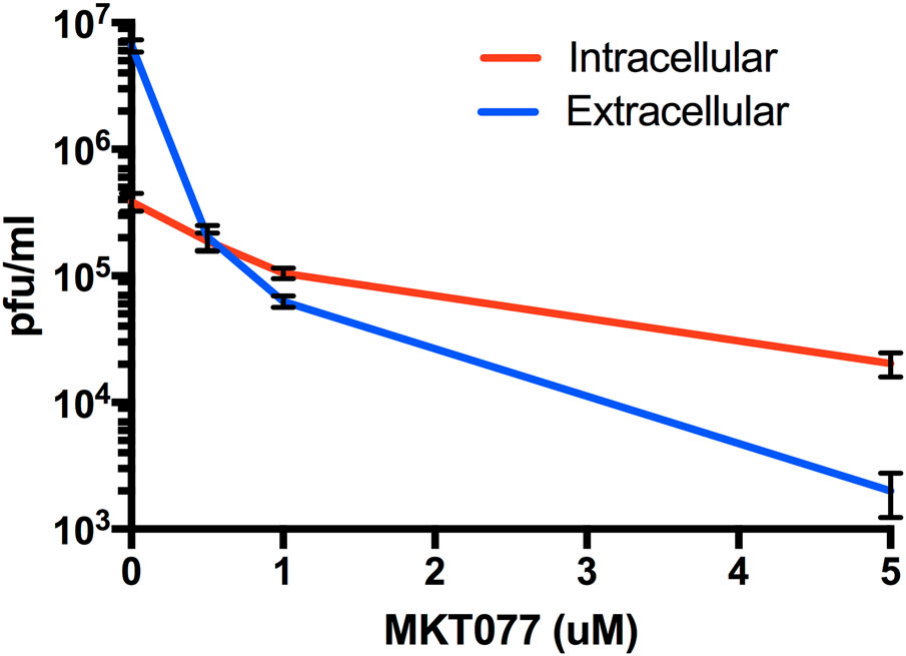
Role of Hsp70 in ZIKV release step. Titres of intra- and extra-cellular virus production in the presence or absence of MKT077 are shown. Following pre-treatment with 0.5, 1, and 5 µM MKT077, Huh7.5 cells were infected with ZIKV (0.1 MOI. Free virus (supernatants) and cell-associated viruses were collected 48-h post-infection. Infectious viral titres were determined by plaque assay. Error bars denote 95% confidence intervals. *N* = 6 per data point. Error bars denote standard error of the mean. Virus production was significantly different from between intracellular vs. extracellular compartments (*P* < .0001), as well as MKT007 concentration (*P* < .0001). There was a significant interaction term (*P* < .0001) noted by the crossing of the lines at 0.5 µM MKT007.

MKT077 induces accumulation of intracellular ZIKV: We titrated the intracellular vs. extracellular virus titre in MKT007-treated cells to evaluate a possible role of Hsp70 in the release of virus particles. The cell membrane was ruptured by freeze-thaw cycles (3 times) to release the trapped cellular matured and infectious virus particles and was titrated by plaque assay. A dose-dependent increase in the intracellular infectious virus particles was observed compared to the extracellular virus indicating that Hsp70 impacts the release of mature virus particles (Figure 7).

## Discussion

Until recently, ZIKV was not considered a serious public health threat, and thus its biology has not been studied in great detail. Hsp’s have been found to be critical factors for the pathogenesis of many viruses [27]. For example, HIV, rotavirus, and influenza virus replication are negatively regulated by Hsp70, whereas dengue, Japanese encephalitis, hepatitis C and Hazara viruses utilize Hsp70 for replication [17,18,21,28–31]. Here, we have shown that Hsp70 is involved in multiple steps of the ZIKV life cycle ranging from cell entry to replication and virus egress, highlighting its essential role in virus infection.

Previous work identified Axl, Tyro3, and TIM1 as major cellular players in the ZIKV cell entry process. However, Cas9-mediated deletion of Axl-1 in human neuronal progenitor cells and cerebral organoids did not protect these cells from ZIKV infection indicating the multifactorial entry process of the virus [32], and suggesting that ZIKV uses multiple classes of cell surface molecules as receptors in different cell types. As an initial event in the infection process, virus particles attach and aggregate on the cell surface, providing a window of opportunity for the virus to bind to a specific receptor(s) on the dynamic cell membrane. Other molecules (including Hsp70) have been implicated for the attachment and aggregation of other viruses [11,33,34]. Although Hsp70 chaperones do not contain export signal peptide sequences, they are found on the cell surface in clathrin-coated pits and within endosome/lysosome-related vesicles [35]. They translocate spontaneously from the cytosol into the plasma membrane after oligomerization and binding to phosphatidylserine [25].

Our experiments indicate that Hsp70 is involved in ZIKV binding and entry into mammalian cells, highlighting its important role in the early stages of virus infection. We detected the presence of Hsp70 on the cell surface of both monkey and human cells where it could interact with infecting viral particles. Antibody blocking of cell surface-localized Hsp70 or pre-incubation of ZIKV with recombinant Hsp70 significantly reduced ZIKV infection. Specifically, reduction in the viral plaque forming units suggests that initial viral infection was inhibited, consistent with the hypothesis that Hsp70 is one cellular factor acting as a cell surface receptor for ZIKV attachment and invasion.

Hsp70 and related proteins are part of the cellular protective response to stress including the assault from pathogens and cytotoxic agents [36]. Hsp70 also protects cells from reactive oxygen species that cells produce as a part of their antiviral response [37,38]. In viral infections, following entry into the cell, the virus takes over the cellular replication and translation machinery for selfish benefit. In this process, they produce viral mRNA and proteins in excess to maximize production of viral progeny. The virus requires the cellular protein folding machinery for accurate folding and stability of the accumulated superfluous viral protein sequences. Sensing these events, the cell triggers a temporary global protein synthesis shutdown by the unfolded protein response (UPR) and triggers the formation of stress granules (SG) [39,40]. SGs maintain RNA homeostasis under stress conditions by forming dynamic cytoplasmic RNA granules composed of cellular mRNAs and stalled pre-initiation complexes [41]. DENV and WNV are known to inhibit the formation of SG. However, they exploit the SG proteins TIA-1 and TIAR to facilitate the synthesis of viral genomes [42]. ZIKA has been shown to hijack and utilize the G3BP1, TIAR, and Caprin-1 SG proteins to promote its replication and impaired SG assembly and SG formation [43]. Hsp70 not only acts on aggregated or misfolded clients but also on actively disassembled cellular structures. Upon viral entry, translation of the viral polymerase gene (NS5) and replication of the viral genome occurs. Hsp70 chaperones are essential to achieving the native conformation and function of NS5 and for the active replication complex [40]. Interestingly at early timepoints post-infection, a slight decline in Hsp70 expression was noted, possibly because the virus overwhelms the host protein synthesis machinery during that time, but Hsp70 levels were significantly increased 48-h post-infection (Figure 1). Again, viral infections and heat stress lead to transcriptional reprogramming of several cellular pathways by expressing Heat shock factors (HSFs) (transcription factors). For instance, HSF-1 regulates autophagy by modulating the mammalian target of rapamycin kinase 1 (mTORC-1) and SQSTM1/p62-associated proteostasis [44,45]. Flaviviruses including ZIKV utilize the cellular autophagy process for their replication [46]. The dsRNA viral replication intermediate is amplified inside a double membrane vesicle and thus escapes the host cellular antiviral pathway. Co-localization of Hsp70 along with dsRNA indicates similar events during ZIKV infection. Hsp70 is also in association with DNAJB1, which is known to mediate cellular RNA sensing by interacting with MDA5/MAVS [47] that could be validated for ZIKV infection in future studies. Finally, the decreased ratio of extracellular to intracellular infectious virus also demonstrates the role of Hsp70 in the process of virus egress.

Due to the involvement of Hsp70 in the infection processes of multiple viruses, it has been suggested that this protein may be a potential molecular target for antiviral therapies [20,48,49]. In particular, the association between Hsp70 and ZIKV suggests a critical role for this interaction during the viral infection process. Our data suggest that Hsp70 is one of several critical factors mediating the initial entry and post-entry replication and egress of ZIKV. As there is currently no effective therapy or vaccine for ZIKV infection, novel therapies to reduce infection and severe clinical outcomes, particularly for pregnant women and developing fetuses, are of extreme importance. The multiple critical roles of Hsp70 in the infectious life cycle of ZIKV validate Hsp70 as a potential target for future anti-ZIKV therapies.

### Materials and methods

Cells and virus: Human liver (Huh7.5), human embryonic kidney (HEK) 293 T, and monkey kidney (Vero) cells were grown in complete Dulbecco’s modified Eagle’s medium (DMEM), supplemented with 10% fetal bovine serum (FBS; Invitrogen), 1X penicillin and streptomycin (Invitrogen) and 2 mM L-glutamine (Invitrogen). The MR766 ZIKV strain was obtained from BEI Resources, propagated in Vero cells, and titred by standard plaque assays.

Antibodies and chemical reagents: The E glycoprotein of ZIKV was detected in cell lysates using anti-ZIKA E protein antibody, GTX133314 (GeneTex, Inc. USA). Rabbit polyclonal Hsp70 antibody (ab45133) was purchased from Abcam. Rabbit Hsp60 antibody (SAB4501464) was purchased from Sigma. dsRNA antibody (J2 anti-dsRNA Ab) was purchased from SCICONS, Hungary. Alexa 488-conjugated anti-mouse and anti-rabbit antibodies (A11001 and A11034) were purchased from Life Technologies. Goat *β*-actin antibody (ab8229) and rabbit-IgG polyclonal isotype control (ab37415) were purchased from Abcam. Rabbit Axl antibody (C89E7) was purchased from Cell Signaling. MKT077 was purchased from Sigma (M5449).

Immunolocalization of Hsp70: Immunofluorescence microscopy was performed to elucidate the distribution of Hsp70 on mammalian cells. Cells were seeded at a density of 2 × 10^5^ cells/well in a 2-well chamber slide and grown at 37°C with 5% CO_2_. After 24 h, cells were fixed with 4% paraformaldehyde at room temperature and processed for Hsp70 staining. The antibodies used for immunofluorescence included rabbit anti-Hsp70 (1:500) and Alexa Fluor 488–conjugated goat anti-rabbit IgG. CellMask Deep Red Plasma Membrane stain (C10046, Thermofisher) was used for plasma membrane staining. Cells were examined using an Olympus Fluoview 10i-LIV confocal microscope, and images processed using ImageJ software.

Plaque assays: Vero cells (5×10^5^ cells/well) were grown to a confluent monolayer in 6 well plates and infected with serial dilutions of ZIKV-infected culture supernatant. Incubation was carried out for 2 hours at 37°C with 5% CO_2_, after which monolayers were rinsed with sterile phosphate buffered saline (PBS). Monolayers were overlaid with maintenance medium containing 0.6% molten agarose and incubated at 37°C with 5% CO_2_ for 3 days. At the end of incubation period, secondary overlay media (primary overlay media along with 1% neutral red) was poured and incubated at 37°C overnight with 5% CO_2_. The next day plaques were counted.

Plaque reduction neutralization assays: Confluent cell monolayers were grown in 6 well plates and incubated with 5 µg of polyclonal *β*-actin or rabbit IgG, Axl or Hsp70 antibodies at 4°C for 1 h, rinsed with sterile PBS, and incubated with 10^5^ pfu ZIKV particles, then processed as described above for plaque assays.

Western blots: Monolayers of cells were harvested by a cell scraper and pelleted by centrifugation. Cell pellets were washed twice with PBS and lysed in RIPA buffer (Sigma). The lysate was cleared by centrifugation at 14,000 rpm for 20 min at 4°C and subjected to 10% sodium dodecyl sulphate polyacrylamide gel electrophoresis (SDS/PAGE), after which proteins were transferred to nitrocellulose membranes (BioRad) that were blocked for 60 min at room temperature in 1XTBST buffer (50 mM Tris-HCl pH 7.4, 250 mM NaCl, 0.1% Tween-20) containing 5% non-fat milk powder. Membranes were incubated overnight at 4°C with primary antibody (anti-Hsp60 or anti-Hsp70 at 1:1000; anti-EGFP or anti-EGFP at 1:500) primary antibodies. The next day, blots were washed 5 times with 1XTBST and incubated with the corresponding detection antibody (1:2000) in 1XTBST containing 1% milk solution at room temperature for 1 h. Signals were detected with the enhanced chemiluminescence method (GE healthcare) or AP-based colorimetric kit (BioRad).

For Hsp60 and Hsp70 quantitation, blots were scanned and band intensities quantified using ImageJ software (NIH, Bethesda, MD, USA). The background was first subtracted from the gel image, the intensity of the Hsp70 band and Hsp60 control band quantified for each sample by measuring the area under the intensity curve, and the value of Hsp70 divided by the value for Hsp60. Quotients for each sample were normalized to hour 0 for heat treatment or untreated values to calculate protein induction. Uncropped blots are included as Figure S3.

Cell heat shock assay: Confluent cell monolayers were grown in 12 well plates and incubated at 44°C for 20 min or at 37°C (negative control). At 10 h post heat shock, cells were infected with ZIKV at an MOI of 0.1. Culture supernatants were harvested 48 h post-infection and titrated for infectious ZIKV by plaque assay.

Hsp70 overexpression: HEK 293 T cells grown in 24-well tissue culture plates were transfected with 2 µg of EGFP-Hsp70 vector plasmid (Addgene plasmid no 15215) or EGFP the only plasmid using Lipofectamine LTX reagent. At 40-h post-transfection, the culture medium was replaced with 1 ml of fresh DMEM containing 0.1 MOI of the virus and incubated at 37 °C with 5% CO_2_. At 48-h post-infection culture supernatants and cells were harvested. The culture supernatants were titrated for ZIKV by the focus-forming assay. The cell pellets were used to assess the expression of EGFP-Hsp70 and EGFP by the Western blot assay as described above.

MTT assay: To determine cell cytotoxicity, the colorimetric MTT assay (Biovision Inc, USA) was used. Metabolically active, viable cells convert MTT into formazan, a water-insoluble product; however, dead cells lose this ability. Huh7.5 cells (1 × 10^4^ cells/well) were cultured in a 96-well plate at 37°C with 5% CO_2_ and exposed to varying concentrations of MKT077 for 24 h. Cells treated with medium only served as the control group. After removing the supernatant of each well and washing twice by PBS, 20 µl of MTT reagent (5 mg/ml) and 100 µl of medium were introduced. Following incubation for another 3.5 h, the resultant formazan crystals were dissolved in MTT solvent (100 µl) and the absorbance intensity measured by a microplate reader at 600 nm. All experiments were performed in triplicates, and relative cell viability was expressed as a percentage relative to the untreated control cells.

Competition assay: To block virus surface ligands, 10^5^ PFU of ZIKV were incubated with 1000 ng recombinant human Hsp70 (rhHsp70) protein (Enzo Life Science) or BSA (control) for 90 min on ice. Huh7.5 cells were infected with ZIKV preparations at an MOI of 1 at 37°C with 5% CO_2_ for 1 h, after which cells were washed three times with DMEM. Fresh medium was added to infected cells which were then incubated at 48 h at 37°C with 5% CO_2_. As described above, infectious ZIKV particles in the culture supernatant were determined by plaque assay.

Real-time RT-PCR: Huh7.5 cell in 6 well plates were infected with 6 MOI ZIKV and incubated with maintenance medium containing DMSO or MKT077 (5 µm/well). Cells were harvested at an interval of 1, 3, 6 and 9 post-infection. Total cellular RNA from the collected cells was extracted using Direct-Zol RNA purification kits. ZIKV capsid (F- 5’ CAA TCA AGC CAT CAC TGG GC 3’, R- 5’ GCC AAT GAT TCC GAT GCT GG 3’) and host GAPDH (F- 5’ CTC TCT GCT CCT CCT GTT CGA C, R- 5’ TGA GCG ATG TGG CTC GGC T 3’) mRNA was amplified using one-step RT-PCR kit using a Qiagen Rotor Gene real-time PCR machine. REST 2009 Software was used for relative quantification and to analyse ZIKV RNA kinetics.

Statistical analysis: Experiments with paired treatments were analysed by the Mann–Whitney *U* test. Experiments with three treatments were analysed by analysis of variance with Bonnferonni’s correction for multiple tests.
